# Bioactive Compounds Isolated from a Marine Sponge Selectively Inhibit *Neisseria gonorrhoeae*

**DOI:** 10.3390/antibiotics13121229

**Published:** 2024-12-19

**Authors:** Omar E. Christian, Dreyona A. Perry, Alaa I. Telchy, Preston N. Walton, Daniel Williams

**Affiliations:** 1 Department of Chemistry and Biochemistry, North Carolina Central University, Durham, NC 27707, USA; ochristi@nccu.edu; 2 Department of Biological Sciences and Biomedical Sciences, North Carolina Central University, Durham, NC 27707, USA; dperry59@eagles.nccu.edu (D.A.P.); atelchy@eagles.nccu.edu (A.I.T.); 3 Department of Biological Sciences, Alabama State University, Montgomery, AL 36104, USA; pwalton3962@myasu.alasu.edu

**Keywords:** ceftriaxone, sexually transmitted infections, high-level ceftriaxone resistant, natural products, bioactive, minimal inhibitory concentration, antibiotic, metabolite

## Abstract

Background/Objectives: *Neisseria gonorrhoeae* is the third most common sexually transmitted infection (STI), which may become untreatable soon if resistance continues to drastically increase. Due to increases in resistance to recommended antibiotics, alternative sources of novel compounds to combat this threat are being explored. Interestingly, marine sponges have proven to produce a plethora of bioactive compounds that display anticancer, antiviral, antifungal, and antibacterial activity. Methods: In this study, the extracts of the sponge collected from Saint Thomas, US Virgin Islands were examined to determine their antibacterial activity against *E. coli*, *S. aureus*, and *N. gonorrhoeae*. Results: The ethyl acetate sponge extracts significantly inhibited growth of *N. gonorrhoeae*, while none inhibited *S. aureus* and *E. coli*. The bioassay-guided purification of the ethyl acetate extract resulted in the isolation of 6-desmethyl-6-ethylspongosoritin A (1) and plakortone B (2). To determine if the pure sponge metabolite could improve the efficacy of ceftriaxone against a high-level ceftriaxone (HTX)-resistant gonococcal strain, an antibiotic checkerboard assay was done by combining various concentrations of either precursor fractions or the purified compound 2 with ceftriaxone. Plakortone B (2) and ceftriaxone acted in synergy against gonococcal strains and inhibited growth by increasing membrane permeability when exposed for 4 h and 24 h. Conclusions: This suggests that marine sponges may serve as a source for novel bioactive compounds against antibiotic-resistant strains of *N. gonorrhoeae*, as well as improve the efficacy of currently prescribed antibiotics.

## 1. Introduction

*Neisseria gonorrhoeae* (*Ng*) is a Gram-negative bacterium that causes gonorrhea, a sexually transmitted infection (STI) that if left untreated can cause infertility, pelvic inflammatory disease (PID), and increased HIV transmission [1,2]. In 2019, the Center for Disease Control and Prevention (CDC) asserted that *Ng* was an urgent societal threat due to approximately 1.14 million new infections per year and the fact that 550,000 of these cases were estimated to be drug-resistant [3]. Due to increases in antibiotic resistance over several decades, penicillin [4,5,6], tetracycline [6], ciprofloxacin [7], and cefixime [8,9,10,11,12] are no longer recommended as treatment options [13]. A combination of ceftriaxone and azithromycin is currently recommended as a front-line treatment option for gonorrhea [13]. Continued misuse of antibiotics may cause *Ng* to become untreatable and the CDC suggests that even susceptibility to ceftriaxone will likely diminish over time. Consequently, the *Ng* strain H014 isolated in Kyoto, Japan [14] has largely conferred an intermediate level of resistance to cephalosporins, but the accumulation of 13 additional mutations in the *penA* mosaic allele has caused strains to develop a high level of resistance to cephalosporins [10,15,16]. A study demonstrated that transformation of the *penA* mosaic allele into a cephalosporin sensitive *Ng* strain could increase resistance to ceftriaxone and cefixime 300- and 500-fold, respectively [15]. This suggests that resistance to ceftriaxone may be inevitable. Surprisingly, there appears to be a fitness cost associated with antibiotic-resistant strains that can be ameliorated via compensatory mutations [17]. Regardless of the fitness disadvantage associated in vivo for the *penA* mosaic allele [15], the potential dissemination of this mutation and/or other mutations that confer a high level of antibiotic resistance amongst the public is of grave concern. Because of single- and/or multi-dose resistance to antibiotics, there is a need to expand current treatment options via exploring diverse environments for novel compounds against bacteria, like *Ng*. Aquatic biomes constitute 70% of the earth’s surface and contain highly diverse ecosystems that are natural sources of bioactive molecules against cancer and a variety of microorganisms. Marine invertebrates such as sponges produce a plethora of diverse bioactive compounds [18,19,20,21,22,23,24,25,26,27,28] and antibiotics, like manoalide [29]. Caribbean sponges produce a range of bioactive compounds [25,30]. In this study, we wanted to determine if bioactive compounds isolated from *Plakortis* sp. [31,32,33,34,35] inhibit different bacteria and especially, *N. gonorrhoeae*. Plakortone B (**2**) [34,35] selectively inhibited *N. gonorrhoeae* but did not inhibit *E. coli* and *S. aureus*. When compound **2** was used in combination with ceftriaxone, the ceftriaxone-resistant *Ng* strain was more susceptible, which suggests that *Plakortis* sp. may be a source for novel bioactive compounds against ceftriaxone-resistant *N. gonorrhoeae* strains.

## 2. Results

### 2.1. Determining Whether Methanol Sponge Extracts Display Antibacterial Activity

The increasing antibiotic resistance of *Ng* presents a critical challenge to treating gonorrhea, which has necessitated exploration of alternative sources for novel antibacterial compounds. Secondary bioactive metabolites isolated from marine sponge extracts of different geographical regions display a range of antibacterial activity against microorganisms [18,20,24,25]. Therefore, we were interested in determining whether sponge samples collected from the US Virgin Islands (US-VI) displayed antibacterial activity against *Staphylococcus aureus*, *Escherichia coli*, and *N. gonorrhoeae* FA19 wildtype (Table 1). The bioassay-guided purification of the methanolic (MeOH) extract produced seven fractions. The fractions were serially diluted 2-fold in a 96-well antibacterial plate assay. *E. coli* and *S. aureus* were not inhibited within the range of concentrations tested but surprisingly, *N. gonorrhoeae* was inhibited by different concentrations of the sponge extracts. Methanolic fraction 6 (MF-6) displayed the lowest minimum inhibitory concentration (MIC) of ~1 mg/mL for *Ng* after a 4 h and 24 h incubation period (Table 2). Additionally, MF-7 (MIC = 1.56 mg/mL) inhibited *Ng* at 4 h but displayed a similar level of inhibition to MF-6 (~1 mg/mL) at 24 h (Table 2). This suggests that the US-VI marine sponge may contain metabolites that selectively inhibit *N. gonorrhoeae* within the tested concentrations compared to *E. coli* and *S. aureus*.

### 2.2. Antibiotic Profiling of High-Level Resistant Gonococcal Strain

In our preliminary screening (Table 2), *N. gonorrhoeae* FA19_WT_ was the only strain tested that was inhibited by the MeOH sponge extract; therefore, we decide to determine whether an isogenic high-level ceftriaxone resistant *penA*41 mosaic allele strain (HTX-41) was susceptible as well. Although HTX-41 clinical isolates were previously shown to demonstrate a high level of resistance to ceftriaxone [10,15,16,36], we decided to determine whether our laboratory-maintained HTX-41 strain displayed a similar level of resistance. To this end, we determined that HTX-41 was inhibited at 6 μg/mL and 2 μg/mL of ceftriaxone at 4 h and 24 h (Table 3), respectively, which is approximately 75- to 25-fold more resistant than FA19_WT_. Our laboratory-maintained HTX-41 strain displays comparable levels of resistance to strains in the literature [9,15].

### 2.3. Determining Whether EtOAc Sponge Extracts Inhibit Ng Strains

An unfractionated EtOAc extraction (FE) was also tested and shown to inhibit *Ng* strains at lower concentrations for 4 h (MIC = 0.122 and 0.052 mg/mL for FA19_WT_ and HTX-41, respectively) and 24 h (MIC = 0.178 mg/mL and 0.084 for FA19_WT_ and HTX-41, respectively) (Table 4). The FE (MIC = 0.052–0.178, Table 4) was approximately ~6- to 19-fold more effective at inhibiting *Ng* strains than the lowest MeOH sponge extract concentration (MF-6 at an MIC = 1 mg/mL, Table 2). Since the sponge EtOAc extract inhibited *Ng* strains at lower concentrations, this extraction was prioritized for purification by flash chromatography using mixtures of EtOAc/hexane (hx). A total of 6 EtOAc/hexane fractions (A–F, Table 5) were collected and tested for antibacterial activity. Fraction A inhibited FA19_WT_ at 0.059 mg/mL and 0.051 mg/mL for 4 h and 24 h, respectively. It also inhibited the FA19 isogenic mutant HTX-41 strain at an MIC of <0.019 mg/mL, which is lower than the tested concentration range (Table 5). The EtOAc/hx fractions did not inhibit *E. coli* and *S. aureus*; therefore, we decided to focus on identifying which sponge metabolite(s) selectively inhibits *N. gonorrhoeae*.

### 2.4. Determining Whether EtOAc Sponge Subfractions Inhibit Ng Strains

Based on the level of antibacterial activity of unfractionated EtOAc (Table 4) sponge extracts against gonococcal strains, we decided to determine the MIC for *Ng* strains using the subfractionated samples A through F. Strain HTX-41 was more susceptible to multiple fractions (A to F) than the FA19 wildtype (Table 5). The greatest difference in susceptibility of HTX-41 was to fraction A, with an MIC of <0.019 for 4 h and 24 h, and fraction B, with an MIC of 0.110 and <0.086 for 4 h and 24 h, respectively (Table 5). Since HTX-41 showed a lower level of susceptibility to fractions A and B, these extracts were fractionated further using flash chromatography to identify specific secondary metabolites involved in antibacterial activity. Hence, fractions A and B were purified to give AH1 to AH7 (Table 6) and BH1 to BH6 (Table 7). Both the FA19 wildtype and HTX-41 displayed comparable levels of susceptibility that ranged from 0.078 to 0.1 mg/mL for fractions AH4 and AH6 (Table 6). There was a modest increase in susceptibility (MIC of 0.038 to 0.156 mg/mL, Table 7) to subfractions BH3 to BH6 for the strains. This was somewhat surprising given that fraction A inhibited the strains at a lower MIC range of <0.019 to 0.059 compared to fraction B, with an MIC range of <0.086 to 0.340 during the initial screen (Table 5). As shown in Table 7, HTX-41 showed the greatest level of susceptibility to fraction BH5 (MIC of 0.060 mg/mL for 4 h and 0.049 mg/mL for 24 h). Although the MIC trends of BH5 seemed to deviate between 4 h (MIC = 0.046) and 24 h (MIC = 0.156) compared to BH3 and BH4 for FA19 (Table 7), the MIC values, although slightly higher, did not deviate (4 and 24 h MIC = 0.125) in the checkerboard assay (Table 8). There are several factors that could have contributed to the fluctuation of BH5 MIC levels between strains and/or assays. We speculate that the deviation between the 4 h and 24 h MIC values for the FA19 wildtype could have been due to extract aliquot stability (degradation), longer incubation periods negatively affecting component synergy, or the development of strain-specific resistance. Additionally, it is not uncommon for fluctuations in MIC values to occur over longer incubation periods. These factors reflect the complexity of sponge fractions as it relates to determining strain-specific susceptibility. Although BH3 and BH4 demonstrated lower MIC values at 4 h and 24 h, we decided to use BH5 in the checkerboard and permeability assays. BH5 was prioritized over BH3 and BH4 mainly because of comparable MIC levels, especially for HTX-41, given our interest in improving its susceptibility to ceftriaxone and because we had more BH5 available to complete downstream experiments.

### 2.5. Evaluating the Synergistic Interaction of BH5 and Ceftriaxone Against Ng Strains

Because *N*. *gonorrhoeae* is capable of developing resistance to ceftriaxone (TX), the last first-line treatment option [14,16], we wanted to determine whether the marine sponge extract BH5 (Table 7) used in combination with ceftriaxone would increase gonococcal susceptibility. Shown in Table 8 are combinations of BH5 and ceftriaxone in a 2-fold serial dilution checkerboard assay. The assay demonstrated distinct interactions of these compounds against the FA19 wildtype and the isogenic mutant, HTX-41. Combinations of BH5 and ceftriaxone against the FA19 wildtype exhibited synergistic fractional inhibitory concentration indexes (FICI) of 0.469 for 4 h and 0.376 for 24 h. The fraction BH5 and ceftriaxone were also synergistic against HTX-41 with a FICI of 0.416 for 4 h, but additive for 24 h with a FICI of 1.011. The wildtype FA19 strain increased in susceptibility to ceftriaxone when combined with BH5 by approximately 19-fold (TX alone = 0.000038 mg/mL to TX = 0.000002 when combined) compared to a 6.25-fold (TX alone = 0.025 mg/mL to 0.004 mg/mL when combined) increase in susceptibility of HTX-41 during a 4 h incubation period. As previously mentioned, it is possible that longer incubation periods could affect the activity of the sponge extracts, which is why we believe that the BH5 susceptibility increased by approximately 7.5-fold (TX alone = 0.000015 mg/mL to 0.000002 mg/mL combined) for the FA19 wildtype for 24 h. Regardless of whether a longer incubation period affected the sponge extract’s activity, we still observed a 91-fold increase in susceptibility of HTX-41 to BH5 during a 24 h incubation period. It is noteworthy to mention that the large increase in susceptibility of HTX-41 is most likely additive (Table 8) against the strain HTX-41. Nevertheless, HTX-41 appears to be more susceptible to either the combination and/or concentration of these compounds during longer incubations. It is possible that BH5 may increase susceptibility of HTX-41 through an unknown mechanism.

### 2.6. Examination of the Impact of BH5 on Bacterial Membrane Permeability

**Figure 1:**
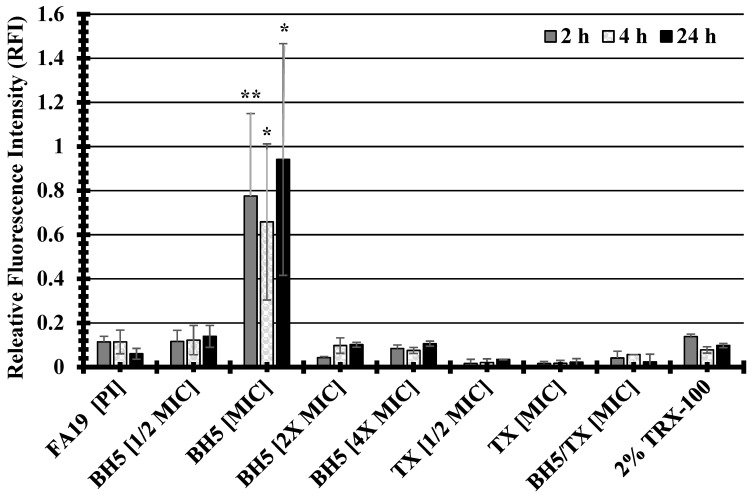
The compound BH5 isolated from a marine sponge increases membrane permeability of the FA19 wildtype. P values were determined via one-way ANOVA and are represented via asterisks to indicate levels of significance: (*) *p* < 0.05; (**) *p* < 0.01. The standard error of the mean (SEM) was determined based on assays using four independent biological samples.

A previous study demonstrated that bioactive metabolites extracted from the sea sponge, *Discodermia kiiensis*, permeabilized the plasma membrane of cells to non-permeable reagents by interacting with plasma membrane phospholipids [27]. To determine whether the sponge extract BH5 was capable of permeabilizing the bacterial cell membrane of the FA19 wildtype, propidium iodide (PI) was used in a 96-well plate membrane permeability assay. Propidium iodide does not penetrate intact membranes but when membranes become damaged, PI enters and produces a fluorescence that can be measured as relative fluorescence intensity (RFI) to that of untreated cells. Because the FA19 wildtype and HTX-41 are genetically identical except for mutations in the HTX-41 *penA* allele, which confers high-level ceftriaxone resistance, we did not test for its changes in membrane permeability. As indicated in Figure 1, the FA19 wildtype demonstrated an increase in membrane permeability when cells were treated with BH5 at an MIC = 0.04–0.06 mg/mL during 2 h, 4 h, and 24 h incubations compared to untreated cells. A 6.8-fold increase in the RFI of BH5-treated (RFI = 0.7775) compared to untreated (RFI = 0.1143) gonococcal cells was determined for 2 h. The RFI of treated gonococcal cells (RFI = 0.6582) at 4 h was approximately 6-fold more than that determined for untreated (RFI = 0.1143) cells. There was a 15.6-fold increase in the RFI for treated (RFI = 0.9413) compared to untreated cells (RFI = 0.0604) after 24 hrs. Although the high RFI at 24 h for the FA19 wildtype (Figure 1) could be viewed as ambiguous given the deviation in BH5 MIC levels (from 4 h MIC = 0.046 to 24 h MIC = 0.156, Table 7), which suggests bacterial recovery, there are other possibilities as to why there was a difference between membrane permeability and MIC levels. The BH5 MIC (0.156) at 24 h appeared to fluctuate (see Table 7), but when BH5 was used in the checkerboard assay (see Table 8) a higher (4 h and 24 h MIC = 0.125) but consistent MIC value was produced. Regardless of the MIC ambiguity at 24 h (Table 7), the high RFI (Figure 1) could be due to stability, diffusion, or interaction of specific active components with the bacterial membrane over time in different growth media. Thus, specific active components might be less effective at inhibiting growth (higher MIC) but still capable of disrupting cell membranes (lower MIC) at lower concentrations depending on the growth environment. Since there was a significant (*p* values < 0.05; refer to Figure 1) increase in the RFI for treated cells at an MIC = 0.046 mg/mL during different incubation times, this suggests that BH5 impairs the cell membrane through an unknown mechanism.

### 2.7. Structure Elucidation of 6-Desmethyl-6-Ethylspongosoritin A (***1***) and Plakortone B (***2***)

**Figure 2:**
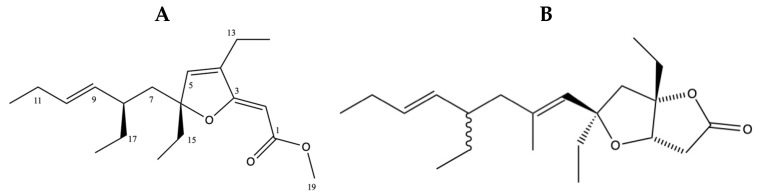
Structures of (**A**) 6-desmethyl-6-ethylspongosoritin A (**1**) and (**B**) plakortone B (**2**).

The fractions AH5_P1 and BH5_P1 (P denotes the fraction number collected from reverse phase chromatography of the fractions AH5 and BH5, Figures S3 and S4) were confirmed as 6-desmethyl-6-ethylspongosoritin A (**1**) by comparison of Figure 2A to the known furano α,β-unsaturated ester metabolites isolated from various *Plakortis* sp. (Table 9; [37,38]), with an average Δδ < 1 ppm. They displayed a [M + H]^+^ 307.22788 in the high-resolution ESI TOF (Figures S5 and S6; Tables S1 and S2), consistent with a molecular formula of C_19_H_30_O_3_. A cursory evaluation of the active fractions AH5_P2 and BH5_P2 suggested that both fractions contained the same active component. AH5_P2 and BH5_P2 displayed a [M + H]^+^ 335.25900 in the high-resolution ESI TOF (Figures S7 and S8; Tables S3 and S4), consistent with a molecular formula of C_21_H_34_O_3_. This is consistent with the known furanolactone, plakortone B (**2**) (Figure 2B) [34,35]. Due to insufficient material, the 1H and 13C NMR of plakortone B (**2**) were not determined. However, the plakortones are a well-established group of metabolites from the Plakortis family that activate cardiac sarcoplasmic reticulum Ca^2+^ ATPase [35] and are cytotoxic [34].

To determine whether each peak displayed a similar level of antibacterial activity, we performed an MIC assay testing both gonococcal strains. In the MIC assay, we determined that BH5_P1 inhibits only one of the strains tested, while BH5_P2 inhibited FA19_WT_ at an MIC of 0.5 mg/mL for both incubation periods and HTX-41 at 0.333 mg/mL for 4 h and 0.5 mg/mL for 24 h (Table 10). Since BH5_P2 inhibited both gonococcal strains, we decided to determine whether it could increase the strains’ susceptibility to ceftriaxone.

### 2.8. Evaluating the Synergistic Interaction of Plakortone B (***2***) and Ceftriaxone Against Ng Strains

The overall focus of this study was to identify specific secondary metabolites that could be used to improve the efficacy of ceftriaxone against *Ng* strains. We showed that the fraction BH5 inhibited the FA19 wildtype and HTX-41 (Table 7), and to a greater extent when combined with ceftriaxone (Table 8). Surprisingly, BH5_P2 was only synergistic with ceftriaxone (FICI = 0.567 for 4 h and FICI = 0.5 for 24 h) against the FA19 wildtype (Table 10). The susceptibility of the FA19 wildtype increased from 0.0009 mg/mL to 0.0000625 mg/mL (14-fold) when BH5_P2 (0.25 mg/mL) and ceftriaxone (0.0000625 mg/mL) were combined for 4 h and an unexpected 2000-fold (0.01 mg/mL of TX to 0.0000047 mg/mL) increase in susceptibility for 24 h (Table 11). The antibacterial effects of BH5_P2 and ceftriaxone against HTX-41 were additive at different concentrations and incubation periods (Table 11). Although the compound combinations against HTX-41 were only additive (FICI ranged from 1 to 1.3), there was an approximate 63- to 129-fold increase in susceptibility to ceftriaxone during a 4- and 24-h exposure period, respectively. This still suggests that it may be possible to use plakortone B (**2**) to improve the efficacy of ceftriaxone against gonococcal strains.

### 2.9. The Impact of AH5_P2 on Bacterial Membrane Permeability

**Figure 3:**
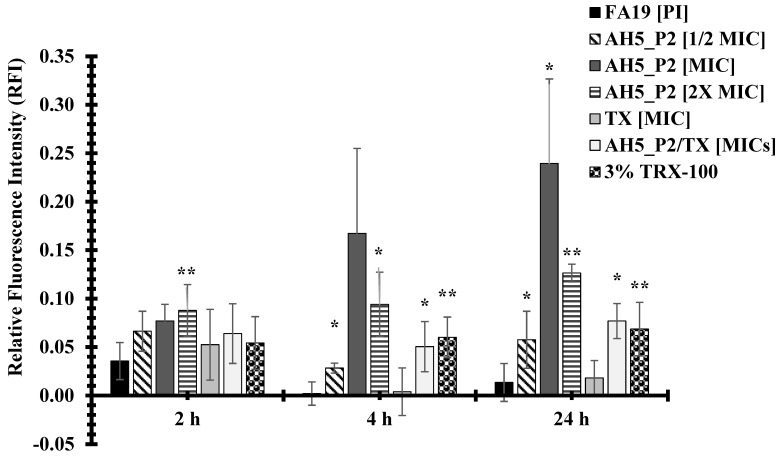
Compound AH5_P2 isolated from a marine sponge extract AH5 increases the membrane permeability of the FA19 wildtype. P values represented via asterisks indicate levels of significance: (*) *p* < 0.05; (**) *p* < 0.01. The standard error of the mean (SEM) was determined based on assays using four independent biological samples.

**Figure 4:**
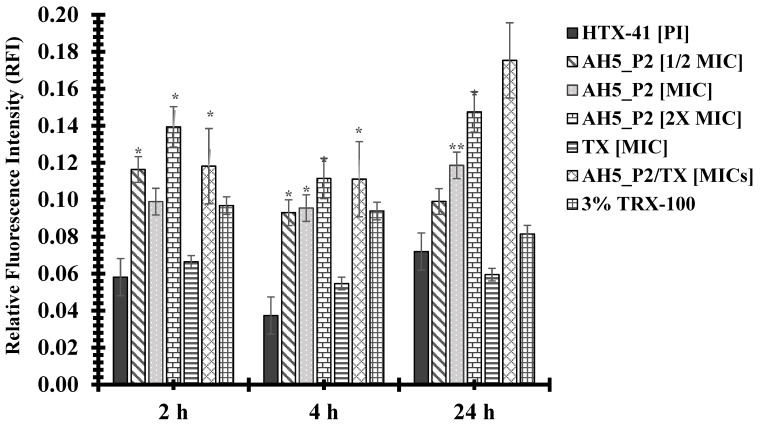
Compound AH5_P2 increases the membrane permeability of strain HTX-41. P values represented via asterisks indicate levels of significance: (*) *p* < 0.05; (**) *p* < 0.01. The standard error of the mean (SEM) was determined based on assays using three independent biological samples.

The compounds AH5_P2 and BH5_P2 displayed similar levels of antibacterial activity against both gonococcal strains (Table 10) but because of limited BH5_P2 material, we had to perform permeability assays with compound AH5_P2. Interestingly, NMR and spectral analysis (Figure S1) revealed that AH5_P1/BH5_P1 and AH5_P2/BH5_P2, respectively, were identical compounds. Although the BH5 sponge extract displayed lower MICs (Table 7) when initially tested than the AH5 sponge extract (Table 6), we were surprised when AH5_P2 (isolated from the AH5 extract) displayed lower MICs than BH5_P2 (isolated from the BH5 extract) (Table 10). The FA19 wildtype was approximately 2.9-fold (MIC = 0.175 mg/mL) more susceptible to AH5_P2 than BH5_P2 (MIC = 0.5 mg/mL), whereas HTX-41 was 5.6-fold more susceptible to AH5_P2 (MIC = 0.066 mg/mL) than BH5_P2 (MIC = 0.333 mg/mL) at 4 h incubation. Also, both strains were 3.8-fold more susceptible to AH5_P2 (MIC = 0.131 mg/mL) than BH5_P2 (MIC = 0.5 mg/mL) at 24 h incubation, which indicates that AH5_P2 displays greater antibacterial activity than BH5_P2. As indicated in Figure 3, we show that compound AH5_P2 affects the membrane permeability of the FA19 wildtype, determined by the changes in RFI due to the entry of PI. The most significant effect on membrane permeability was at the 4 h and 24 h incubation periods for a range of AH5_P2 concentrations. Although several concentrations of AH5_P2 appeared to affect membrane permeability, the 2× MIC (MIC = 0.175 mg/mL, Table 10) showed a similar effect on the FA19 wildtype membrane permeability (*P* > 0.05) (Figure 3). We also determined that membrane permeability increased at 4 h and 24 h when AH5_P2 at an MIC of 0.175 mg/mL was combined with ceftriaxone (TX) at an MIC of 0.00007 mg/mL (Figure 3). It is possible that combining AH5_P2 with TX contributes to a general weakening of the cell envelope, which may indirectly contribute to increases in membrane permeability. With respect to HTX-41, we show a similar change in membrane permeability when AH5_P2 was used at MIC (0.066 mg/mL) for 4 h and 24 h (Figure 4). When cells were treated with 2X MIC, we observed an increase in membrane permeability for each incubation period that was consistent with increases in the FA19 wildtype membrane permeability (Figure 2). Like the FA19 wildtype, the membrane permeability of HTX-41 increased when AH5_P2 and TX were combined at their respective MIC concentrations (Figure 4). Based on these results, AH5_P2 increases membrane permeability at the MIC and 2× MIC for gonococcal strains with different susceptibility levels to ceftriaxone.

## 3. Discussion

In this study, we highlight the urgent need for novel antibacterial compounds to combat the increasing level of antibiotic resistance of *Ng*, which indicates that this issue has become a public health concern. *Ng* has become resistant to several antibiotics that are no longer recommended treatment options [39]. This is further supported by global surveillance data indicating increases in widespread antibiotic resistance [40], specifically to ceftriaxone, a current frontline treatment option [41]. For example, the high level of resistance displayed by *Ng* strain H041, from Kyoto, Japan, is due to multiple mutations accumulated in the *penA* mosaic allele [42]. Transformation of the *penA* allele into sensitive strains also highlights the potential for rapid dissemination of antibiotic resistance [43]. Therefore, the use of marine sponges from the US Virgin Islands as a source of bioactive compounds against *N. gonorrhoeae* may assist in combating resistance.

Studies have shown that marine sponges produce a plethora of bioactive compounds with antibacterial properties [19,20,21,22,28]. For instance, secondary metabolites from marine sponges are able to inhibit different bacterial strains [19,44]. Herein, we demonstrate that ethyl acetate sponge extracts significantly inhibit *Ng* but not *E. coli* and *S. aureus*, which suggests that these compounds may be selective against *Ng*. Thus, we showed that plakortone B (**2**) isolated from the active fractions AH5_P2 and BH5_P2 (identical compounds based on mass spectral data, Figures S7 and S8 and Tables S3 and S4) can inhibit *Ng* strains alone or when combined with ceftriaxone. These findings are noteworthy, especially because susceptibility increased by approximately 2000-fold for the FA19 wildtype strain to plakortone B (**2**) and ceftriaxone. Similar results were observed in a study where secondary plant metabolites significantly lowered the MIC of antibiotics against resistant strains [45]. Although our compound combinations were additive against the HTX-41 *Ng* strain, overall susceptibility did increase, which seems to parallel other studies where either ceftriaxone and/or other antibiotics were used in combination with different natural products [46,47]. Plakortone B (**2**) disrupted *Ng* membrane permeability, especially when combined with ceftriaxone, aligning with other findings indicating that secondary metabolites can disrupt bacterial membranes [45,47]. Similarly, plakortone B (**2**) was isolated from the Chines marine sponge *Plakortis simplex*, which produces a range of cytotoxic furanolactones [48]. Sato et al. suggest that discodermin A, a novel bioactive peptide, apparently interacts with the plasma membrane via phospholipids to reduce membrane integrity [27]. We believe that plakortone B (**2**) could possibly be interacting with phospholipids in a similar manner. It is also worth noting that plakortone B (**2**) and 6-desmethyl-6-ethylspongosoritin A (**2**) have a lactone ring structure like that found in certain antibacterial and antifungal compounds that are known to disrupt cell membranes and cell walls [34,35,49,50]. The observed synergistic and/or additive effects of plakortone B (**2**) resulting in increased membrane permeability are consistent with the idea that the use of secondary metabolites combined with certain antibiotics can increase antibiotic efficacy.

## 4. Materials and Methods

### 4.1. Collection and Isolation

The sponge specimen (07017) was collected by SCUBA in the waters off Brewers Bay Beach, St. Thomas US-VI in 2007. A voucher specimen was deposited in the Department of Chemical and Physical Sciences at the University of the Virgin Islands. The dried macerated sponge material (500 g) was sequentially extracted with hexane (hx), ethyl acetate (EtOAc), and methanol (MeOH) (Figure S2). The solvents were removed in vacuo to afford the crude organic extracts. A portion of the EtOAc extract (20.0 g) was absorbed unto silica gel and subjected to flash chromatography using a EtOAc–hexane gradient (0–100%). Several fractions were collected and used in subsequent assays. Moreover, the subfractions AH5 and BH5 were subjected to repeated normal phase chromatography, eluting with mixtures of EtOAc–hexane, followed by reversed phase chromatography to yield the compounds P1 and P2 (Figure S2).

### 4.2. NMR Analysis of Sponge Components

All 1D and 2D NMR spectra were recorded in CDCl3 on a Bruker AVANCE III NMR spectrometer at 400 MHz for 1H and 100 MHz for 13C. LCMS was performed on a reversed-phase analytical column (4.6 mm × 250 mm, 5 µm) using a photodiode array (PDA) detector and with an electrospray single quadrupole mass spectrometer. High-resolution mass measurements were obtained on an Agilent 6230 ESI-TOF mass spectrometer. The samples were run in positive mode ionization with a capillary voltage of 4000 V. The drying gas (nitrogen) temperature was 325 °C delivered at 10 L/min, and the fragmentation voltage was set to 150 eV. MPLC separation was performed on a Reveleris system equipped with UV and ELSD detectors. All solvents were HPLC grade with 0.1% TFA or ACS grade.

### 4.3. Bacterial Strains and Growth Conditions

The gonococcal strains used in this study (Table 1) were grown on gonococcal (GC) agar plates at 37 °C, 5% CO_2_ for 24 h. Cells were harvested and inoculated into GC broth (GCB) containing Kellogg’s supplements [41], and then shaken at 180 rpm at 37 °C until an optical density (OD 600 nm) of 0.5 (~3.0 McFarland standard) was reached. Cells were diluted to 1.5 × 10^8^ CFU/mL (0.5 McFarland standard) and used in subsequent antibacterial susceptibility assays. All other strains (*E. coli* and *S. aureus*) were grown on Luria Bertani (LB) media at 37 °C overnight and diluted to a McFarland standard equivalent of 1.5 × 10^8^ CFU/mL for antibacterial susceptibility assays.

### 4.4. Antibacterial Susceptibility Assay

To determine whether the sponge extracts/fractions display antibacterial activity against strains and determine the levels of susceptibility of the laboratory-maintained FA19 wildtype and HTX-41 strains to ceftriaxone, a 96-well spot dilution plate assay was done. Briefly, isolated extracts/fractions were serially diluted 2-fold in a 96-well plate and then seeded with 90 μL microliters of GC cells, equivalent to 1.5 × 10^8^ CFU/mL (0.5 McFarland standard). For determining the antibacterial activity of the sponge extracts/fractions, the concentrations ranged from 30 to 0.001 mg/mL depending on the assay. The tested components (sponge extracts/fractions, bacteria, or antibiotics) were dissolved in DMSO. All 96-well plates were incubated at 37 °C, 5% CO_2_ for 4 h and 24 h. Aliquots of 1.5 µL from each well were spotted onto GCB agar plates at the 4 h and 24 h time points. The minimal inhibitory concentration (MIC) was visualized via two independent observers. All assays were done in 3–6 replicates and repeated on three or more independent biological samples.

### 4.5. Membrane Permeability Assays

Gonococcal strains (FA19_WT_ and HTX41) were grown as previously described and then inoculated to an optical density (OD 600 nm) of 0.12 (equivalent to 0.5 McFarland standard, 1 × 10^8^ CFUs). Cells were pelleted at 8000 rpm for 10 min and then resuspended and washed 2× in 10 mL of 5 mM HEPES. One hundred microliters of each strain were removed and seeded in 4 replicates into a Black BD Falcon 96-well plate to serve as controls (minus HEPES). The cell pellets were then resuspended in 20 mL of 5 mM HEPES containing 5 μL of propidium iodide (PI). Afterwards, the cell/PI resuspension was stored in the dark for 10 min before seeding 100 μL of cells from at least three or more replicates into the Black BD Falcon 96-well plate. The plate was agitated for 30 s and then incubated at 37 °C, 5% CO_2_ for 24 h. The FA19 wildtype (½ MIC = 0.025 mg/mL; MIC = ~0.05 mg/mL; 2× MIC = 0.1 mg/mL; and 4× MIC = 0.2 mg/mL) and HTX-41 (½ MIC = 0.03 mg/mL; MIC = 0.06 mg/mL; 2× MIC = 0.12 mg/mL; and 4× MIC = 0.24 mg/mL) were incubated with varying concentrations of BH5 based on the averages of previous MICs determined for 4 h exposures. To determine the effects of combined compounds (BH5 and ceftriaxone, TX) on membrane permeability, FA19_WT_ (0.05 mg/mL of BH5 and 0.00003 mg/mL of TX) and HTX-41 (0.018 mg/mL of BH5 and 0.004 mg/mL of TX) were assayed. These assays were performed as previously described, using a similar 96-well plate layout containing similar controls and an equal number of replicates. The 96-well plates were incubated overnight, and the absorbance was measured at 2 h, 4 h, and 24 h incubation at 37 °C, 5% CO_2_. Absorbance readings were measured at 535 nm excitation and 617 nm emissions over a period of 5 min. All permeability assays were done in replicates of four or more and repeated on three or more independent biological samples to determine the standard error of the mean (SEM) and *p* values.

### 4.6. Checkerboard Assay

Bacterial cultures were prepared as described under the Bacterial Strains and Growth Conditions Section. Briefly, 10 μL of dH_2_O was seeded in each well of a 96-well plate and then combined with 2-fold serially diluted sponge fraction and ceftriaxone. Ninety microliters of GC cells (1.5 × 10^8^ CFU/mL) containing Kellogg’s supplements were inoculated into each well and then incubated at 37 °C, 5% CO_2_ for 18 h. After the incubation, 1.5 μL of each well was spotted on GCB agar plates to determine the fractional inhibitory concentration (FICI) at 4 h and 24 h. The FICI was determined using the following equation: FICI = (MIC of compound 1 and ceftriaxone in combination/MIC of compound 1 alone) + (MIC of ceftriaxone and compound 1 in combination/ceftriaxone alone). The effect from the compound combinations are considered synergistic (FICI ≤ 0.5), additive or indifferent (FICI > 0.5–4), or antagonistic (FICI > 4).

## 5. Conclusions

The rise in antibiotic-resistant *N. gonorrhoeae* has become a health concern, especially considering the continual decrease in treatment options. This study focuses on the potential use of marine sponges, specifically those from Saint Thomas, US Virgin Islands, as a source of novel antibacterial compounds. Interestingly, sponge EtOAc extracts exhibited significant inhibition of gonococcal growth, while not affecting *E. coli* and *S. aureus*. Plakortone B (**2**) was isolated from EtOAc extracts and determined to synergistically enhance the efficacy of ceftriaxone against gonococcal strains by increasing membrane permeability. The synergistic effect suggests that marine sponges are potential sources of novel bioactive compounds to combat evolving and antibiotic-resistant *N. gonorrhoeae* and improve the efficacy of existing antibiotics. This study underscores the importance of exploring diverse environments for novel antimicrobial agents, as the continued increase in antibiotic resistance necessitates the development of new treatment options that lead to more improved public health outcomes.

## Figures and Tables

**Table 1:** Strains used in this study.

Strains	Description	Abbreviation
*N. gonorrhoeae* FA19	wildtype	_WT_
FA19 *penA*41	High level ceftriaxone-resistant strain [9]	HTX-41
*Escherichia coli*	Gram-negative bacteria	*E. coli*
*Staphylococcus aureus*	Gram-positive bacteria	*S. aureus*

**Table 2:** MeOH extracted sponge fractions assayed for antibacterial activity.

Fractions, MF	Minimum Inhibitory Concentration (mg/mL)					
	*N. gonorrhoeae*		*E. coli*		*S. aureus*	
	4 h	24 h	4 h	24 h	4 h	24 h
MF-1	2.93 (±0.7)	1.56 (±0.3)	NI	NI	NI	NI
MF-2	6.25 (±0.0)	5.86 (±0.4)	NI	NI	NI	NI
MF-3	2.95 (±0.1)	2.04 (±0.2)	NI	NI	NI	NI
MF-4	4.04 (±0.5)	2.6 (±0.2)	NI	NI	NI	NI
MF-5	7.55 (±1.1)	4.69 (±0.5)	NI	NI	NI	NI
MF-6	1.17 (±0.1)	1.04 (±0.1)	NI	NI	NI	NI
MF-7	1.56 (±0.0)	0.98 (±0.1)	NI	NI	NI	NI

N = 3; replicates = 4.

**Table 3:** Susceptibility of the gonococcal strains, FA19 wildtype and HTX-41, to ceftriaxone.

Time (h)	Minimum Inhibitory Concentration (μg/mL)	
	FA19_WT_	HTX-41
4	0.08	6
24	0.08	2

N = 3; replicates = 4.

**Table 4:** Unfractionated EtOAc sponge extract displays antigonococcal activity.

Time (h)	Minimum Inhibitory Concentration (mg/mL)	
	FA19_WT_	HTX-41
4	0.122	0.052
24	0.178	0.084

N = 3; replicates = 4.

**Table 5:** Antigonococcal activity of EtOAc/hx sponge fractions.

Fractions	Minimum Inhibitory Concentration (mg/mL)			
	FA19_WT_		HTX-41	
	4 h	24 h	4 h	24 h
A	0.059	0.051	<0.019	<0.019
B	0.34	0.287	0.110	0.086
C	0.115	0.074	0.085	0.077
D	0.277	0.235	0.230	0.245
E	0.434	0.244	0.2	0.175
F	0.246	0.292	0.230	0.170

N = 3; replicates = 4.

**Table 6:** Antigonococcal activity of subfractions (AH1–AH7) of the EtOAc/hexane fraction A.

Fractions	Minimum Inhibitory Concentrations (mg/mL)			
	4 h		24 h	
	FA19_WT_	HTX-41	FA19_WT_	HTX-41
AH1	NI	15	NI	15
AH2	NI	35	NI	35
AH3	NI	1.2	NI	35
AH4	0.1	0.078	0.082	0.078
AH5	0.136	0.196	0.136	0.152
AH6	0.078	0.1	0.078	0.095
AH7	0.781	0.416	0.729	0.494

NI, not inhibited; N = 3; replicates = 4.

**Table 7:** Antigonococcal activity of subfractions (BH1–BH7) of the EtOAc/hexane fraction B.

Fractions	Minimum Inhibitory Concentrations (mg/mL)			
	4 h		24 h	
	FA19_WT_	HTX-41	FA19_WT_	HTX-41
BH1	NI	NI	NI	NI
BH2	NI	NI	NI	NI
BH3	0.038	0.052	0.074	0.069
BH4	0.037	0.043	0.052	0.046
BH5	0.046	0.060	0.156	0.049
BH6	0.208	0.156	0.174	0.156

NI, not inhibited; N = 3; replicates = 4.

**Table 8:** Antigonococcal activity of fraction BH5 and ceftriaxone.

Time (h)	Strains	Agent	MIC (mg/mL)		FIC	FICI	Interpretation
			Alone	Combined			
4	FA19_WT_	BH5	0.125	0.0521	0.417	0.469	Synergy
		TX	0.000038	0.000002	0.053		
	HTX-41	BH5	0.072	0.019	0.26	0.416	Synergy
		TX	0.025	0.004	0.155		
24	FA19_WT_	BH5	0.125	0.0313	0.250	0.376	Synergy
		TX	0.000015	0.000002	0.125		
	HTX-41	BH5	0.109	0.000063	1	1.011	Additive
		TX	0.00283	0.000031	0.011		

WT, wildtype; TX, ceftriaxone; HTX, high level TX resistance; synergy ≤ 0.5; additive > 0.5–4.0; N = 3; replicates = 4.

**Table 9:** Comparison of the ^13^CNMR data of 6-desmethyl-6-ethylspongosoritin A with the literature [37,38].

	Experimental	Theoretical
1	167.0	166.0
2	83.3	84.4
3	172.2	171.5
4	140.3	139.9
5	139.3	139.7
6	97.8	97.2
7	43.5	43.6
8	39.7	39.9
9	134.0	133.5
10	132.1	131.9
11	25.6	25.9
12	14.0	14.1
13	18.5	18.7
14	11.8	11.9
15	32.3	32.4
16	8.0	7.9
17	29.4	29.6
18	11.4	11.5
19	50.5	50.1

**Table 10:** Secondary metabolites isolated from AH5 and BH5 display antigonococcal activity.

Fractions	Minimum Inhibitory Concentrations (mg/mL)			
	4 h		24 h	
	FA19_WT_	HTX-41	FA19_WT_	HTX-41
AH5_P1	NI	1.75	NI	1.75
AH5_P2	0.175	0.066	0.131	0.131
BH5_P1	NI	NI	NI	NI
BH5_P2	0.5	0.333	0.5	0.5

NI, not inhibited; N = 3; replicates = 4.

**Table 11:** Antigonococcal activity of BH5_P2 and ceftriaxone (TX) after a 4 h and 24 h exposure.

Time (h)	Strain	Agent (mg/mL)	MIC (mg/mL)		FIC	FICI	Interpretation
			Alone	Combined			
4	FA19_WT_	BH5_P2	0.5	0.25	0.5	0.567	Synergy
		TX	0.0009	0.0000625	0.067		
		BH5_P2	0.5	0.375	0.75	0.783	Additive
		TX	0.0009	0.0000313	0.033		
	HTX	BH5_P2	0.5	0.5	1	1.016	Additive
		TX	0.004	0.000063	0.016		
		BH5_P2	0.5	0.5	1	1.008	Additive
		TX	0.004	0.000031	0.008		
24	FA19_WT_	BH5_P2	0.5	0.25	0.5	0.5	Synergy
		TX	0.01	0.0000047	0.0003		
		BH5_P2	0.5	0.5	1	1	Additive
		TX	0.01	0.0000023	0.00015		
	HTX	BH5_P2	0.375	0.5	1.33	1.3	Additive
		TX	0.004	0.000065	0.016		
		BH5_P2	0.375	0.5	1.33	1.3	Additive
		TX	0.004	0.0000313	0.008		

WT, wildtype; TX, ceftriaxone; HTX, high level TX resistance; synergy ≤ 0.5; additive > 0.5–4.0; N = 3; replicates = 4.

## Data Availability

The original contributions presented in this study are included in the article/Supplementary Materials. Further inquiries can be directed to the corresponding author.

## Supplementary Materials

The following supporting information can be downloaded at https://www.mdpi.com/article/10.3390/antibiotics13121229/s1. Figure S1. NMR Spectra. An NMR spectrum was determined for the secondary metabolite AH5 P1 based on (A). 1H and (B). 13C NMR of AH5 P1. Figure S2. Isolation of bioactive metabolites from Plakortis. Figure S3. HPLC chromatogram of 07017XFE AH5 showing the elution of P1 at 10.5 min and P2 at 12.5 min. Figure S4. HPLC chromatogram of 07017XFE BH5 showing the elution of P1 at 12.5 min and P2 at 13.5 min. Figure S5. High resolution mass spectral chromatogram (HRMS) of AH5 P1. Figure S6. High resolution mass spectral chromatogram (HRMS) of BH5 P1. Figure S7. High resolution mass spectral chromatogram (HRMS) of AH5 P2. Figure S8. High resolution mass spectral chromatogram (HRMS) of BH5 P2. Table S1. Molecular formula generated from mass spectrometry of AH5 P1. Table S2. Molecular formula generated from mass spectrometry of BH5 P1. Table S3. Molecular formula generated from mass spectrometry of AH5 P2. Table S4. Molecular formula generated from mass spectrometry of BH5 P2.
