# Supplementary material for: Bioactive Compounds Isolated from a Marine Sponge Selectively Inhibit Neisseria gonorrhoeae
Source: Antibiotics (Basel). 2024 Dec 19;13(12):1229. doi: 10.3390/antibiotics13121229 (PMC11726862; doi:10.3390/antibiotics13121229)
Supplement: Supplementary file 1 [file antibiotics-13-01229-s001.zip › antibiotics-3340500-supplementary.pdf]

Supplementary Materials.

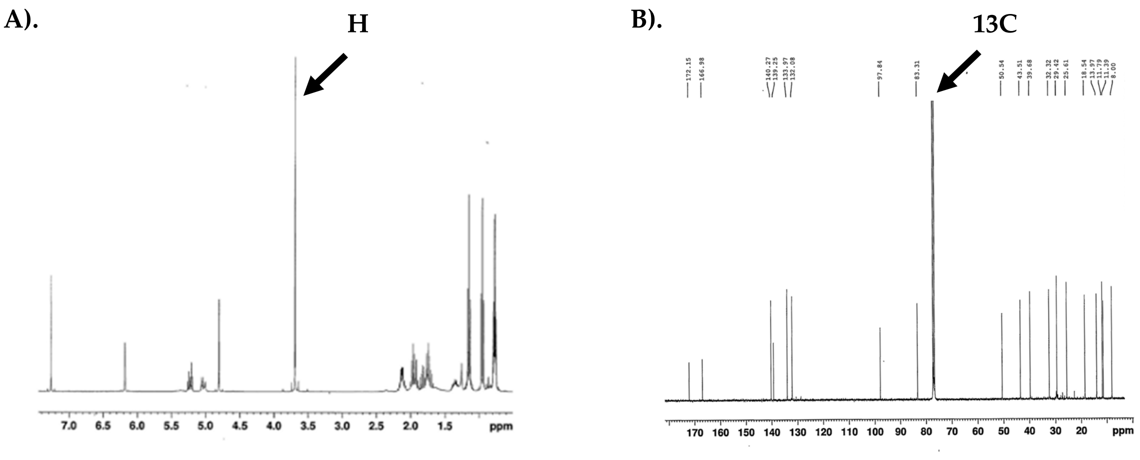

Figure S1. NMR Spectra. An NMR spectrum was determined for the secondary metabolite, AH5 P1 based on (A). 1H and (B). 13C NMR of AH5 P1.

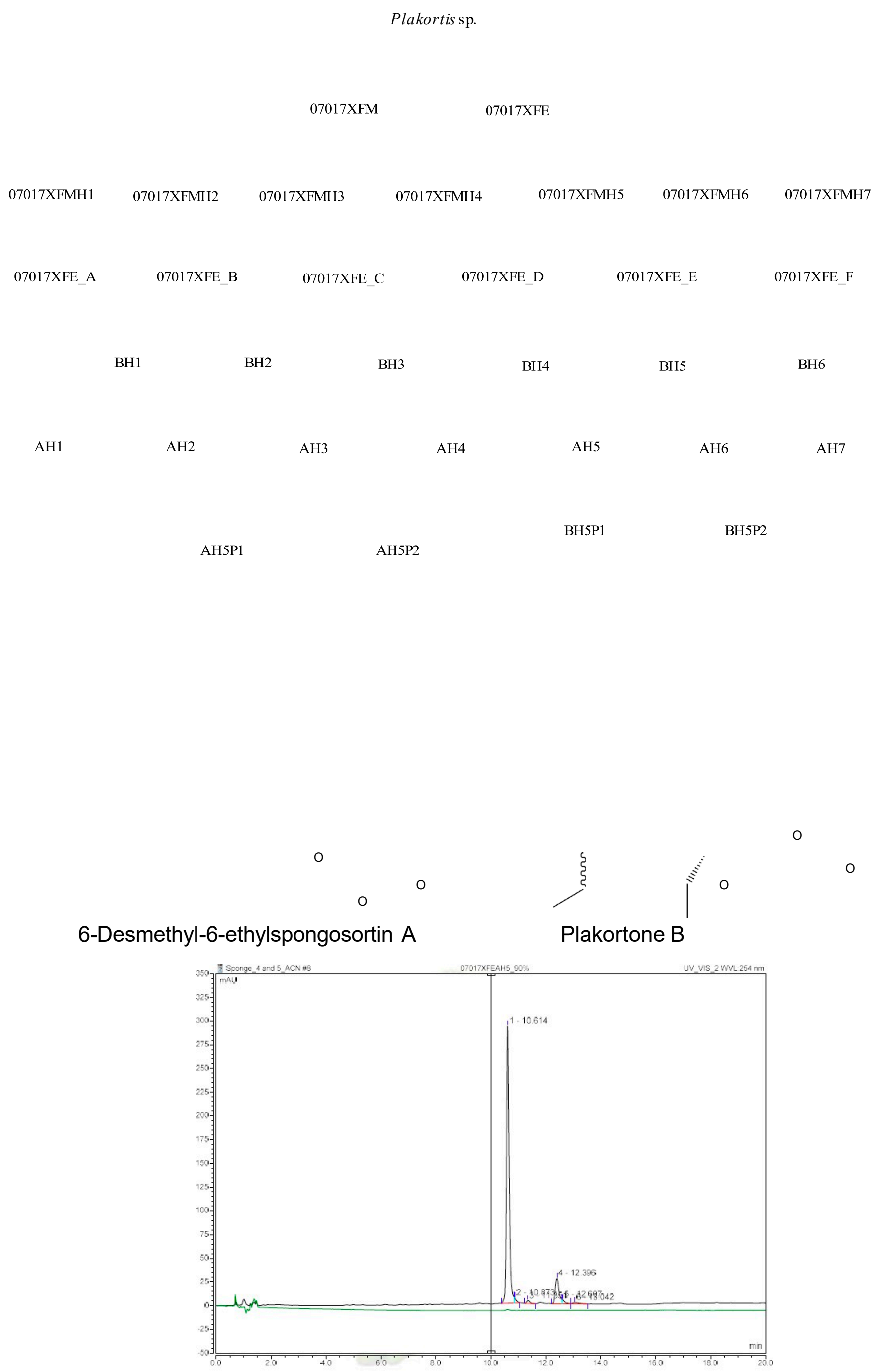

Figure S2. Isolation of bioactive metabolites from *Plakortis*.

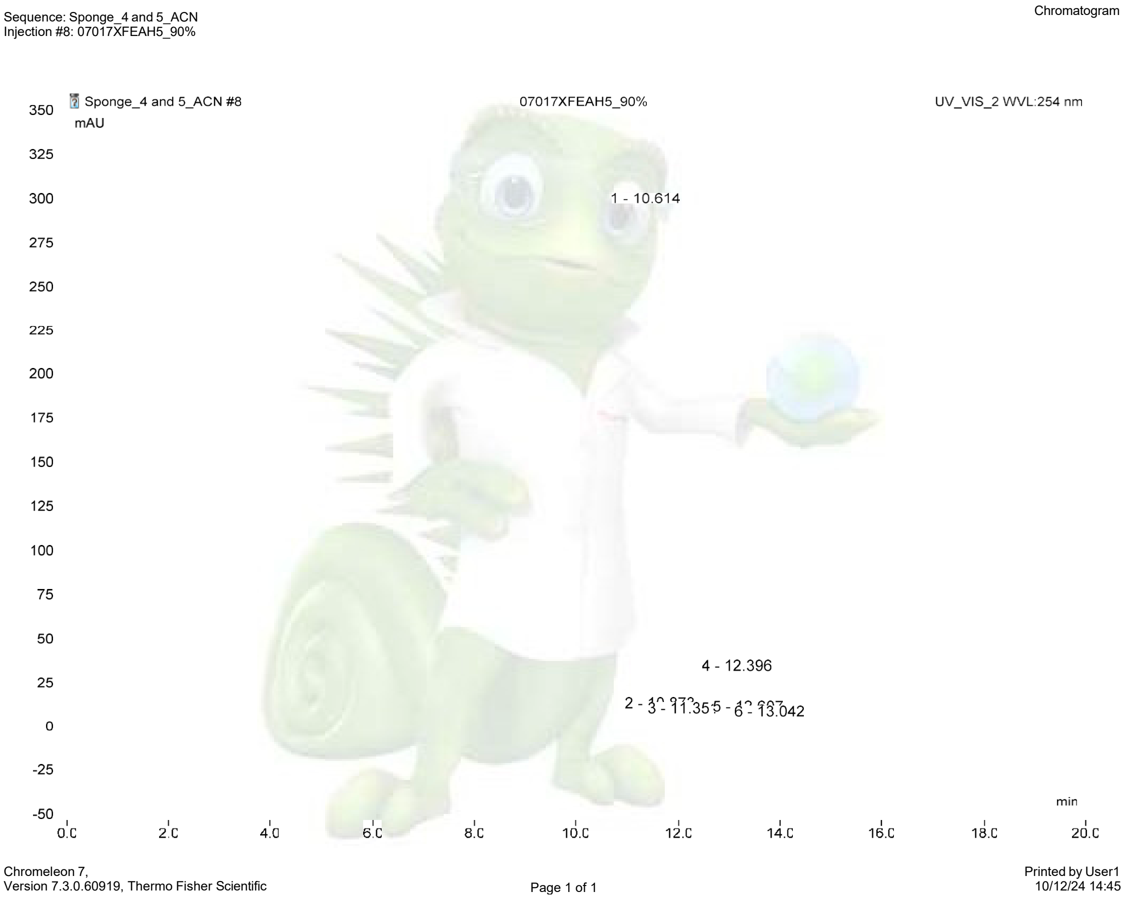

Figure S3. HPLC chromatogram of 07017XFE AH5 showing the elution of P1 at 10.5 mins and P2 at 12.5 mins.

Sequence: Sponge_4 and 5_ACN
Injection #5: 07017XFEBH5

Chromatogram

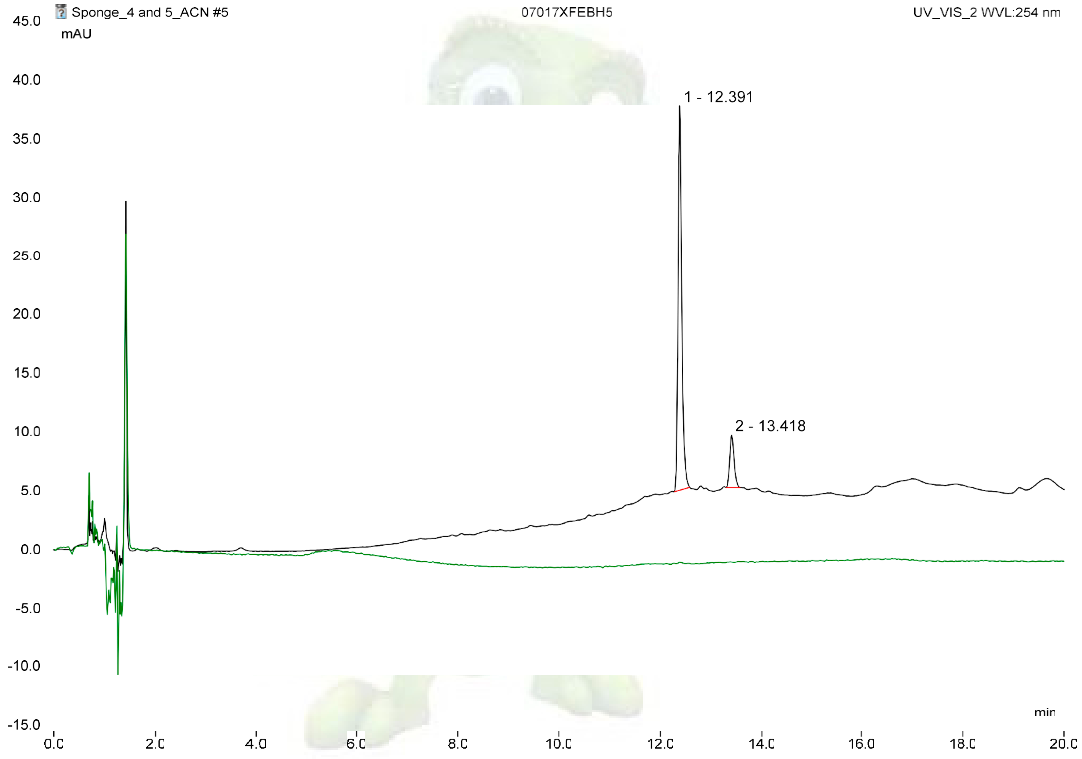

Chromeleon 7,
Version 7.3.0.60919, Thermo Fisher Scientific

Page 1 of 1

Printed by User1
10/12/24 14:48

Figure S4. HPLC chromatogram of 07017XFE BH5 showing the elution of P1 at 12.5 mins and P2 at 13.5 mins.

Figure S5. High resolution mass spectral chromatogram (HRMS) of AH5 P1.

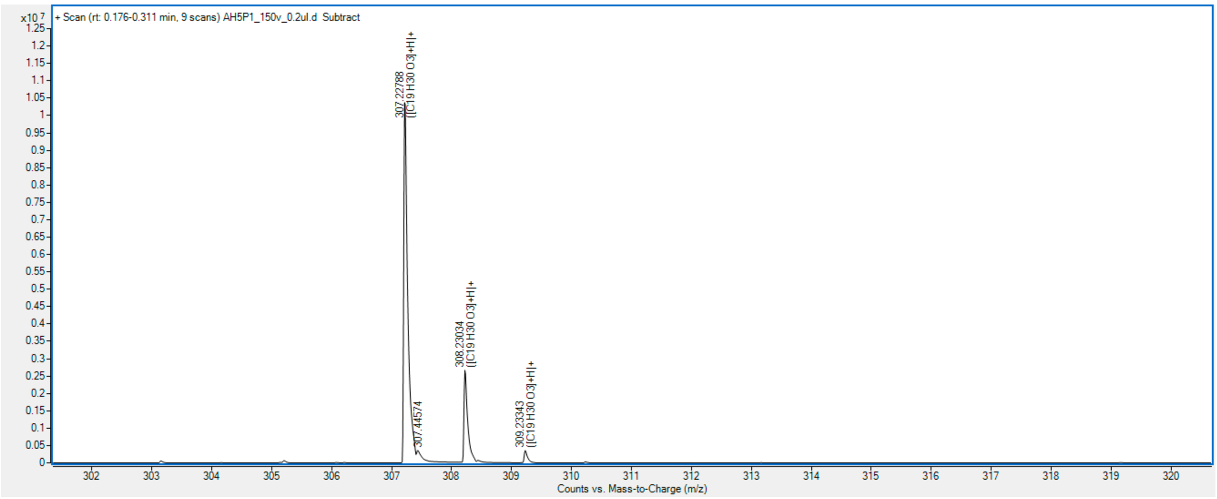

Table S1. Molecular formula generated from mass spectrometry of AH5 P1.

Formula	Species	m/z	Score	Diff(abs. ppm)	Mass
C19 H31 O3	(M+H)+	307.22788	92.29	2.95	306.21949

Figure S6. High resolution mass spectral chromatogram (HRMS) of BH5 P1.

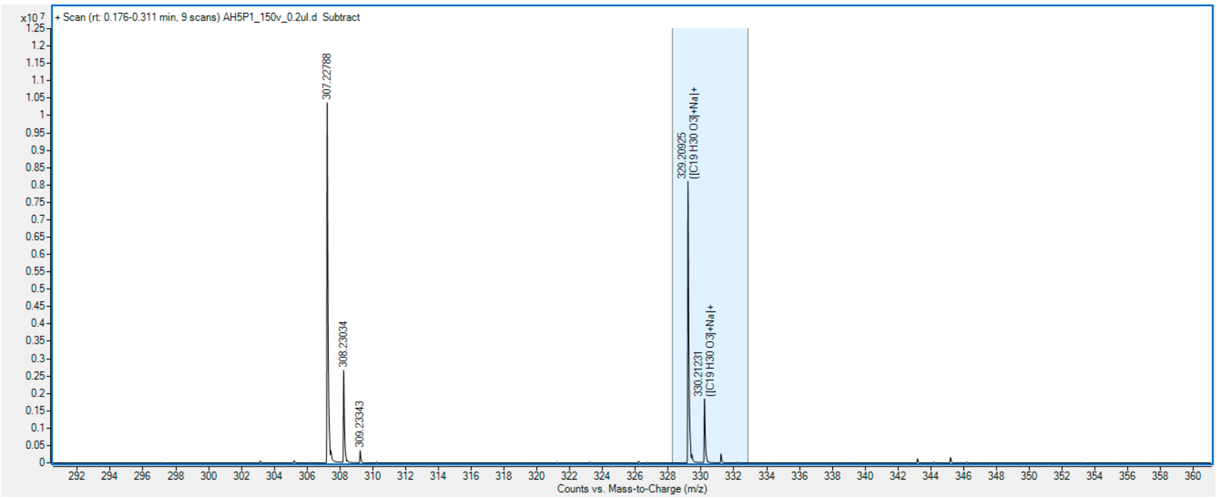

Table S2. Molecular formula generated from mass spectrometry of BH5 P1.

Formula	Species	m/z	Score	Diff(abs. ppm)	Mass
C19 H31 O3	(M+H)+	307.22788	92.29	2.95	306.21949

Figure S7. High resolution mass spectral chromatogram (HRMS) of AH5 P2.

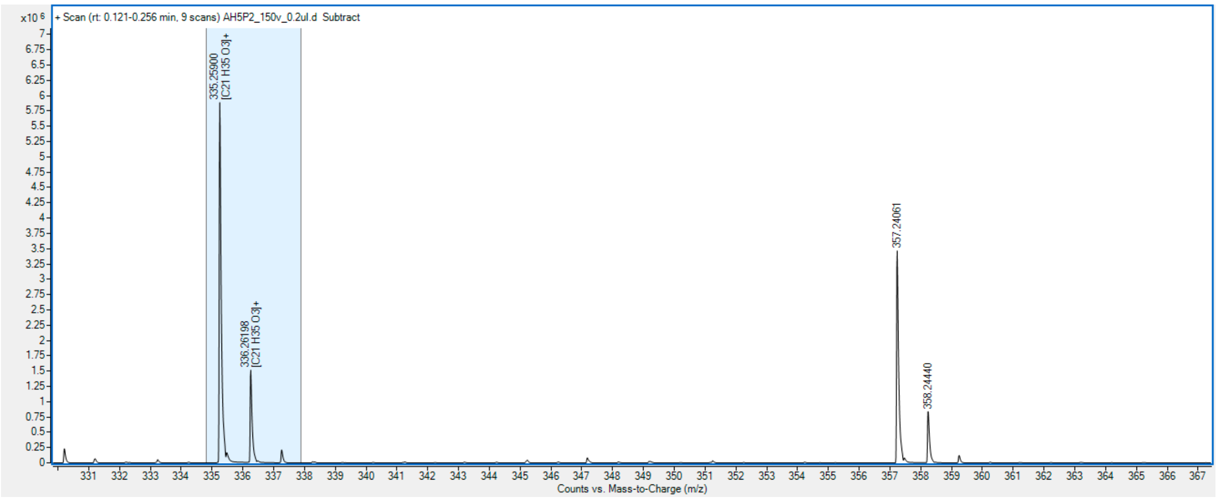

Table S3. Molecular formula generated from mass spectrometry of AH5 P2.

Formula	Species	m/z	Score	Diff(abs. ppm)	Mass
C21 H34 O3	(M+H)+	335.25900	96.36	2.5	334.25079

Figure S8. High resolution mass spectral chromatogram (HRMS) of BH5 P2.

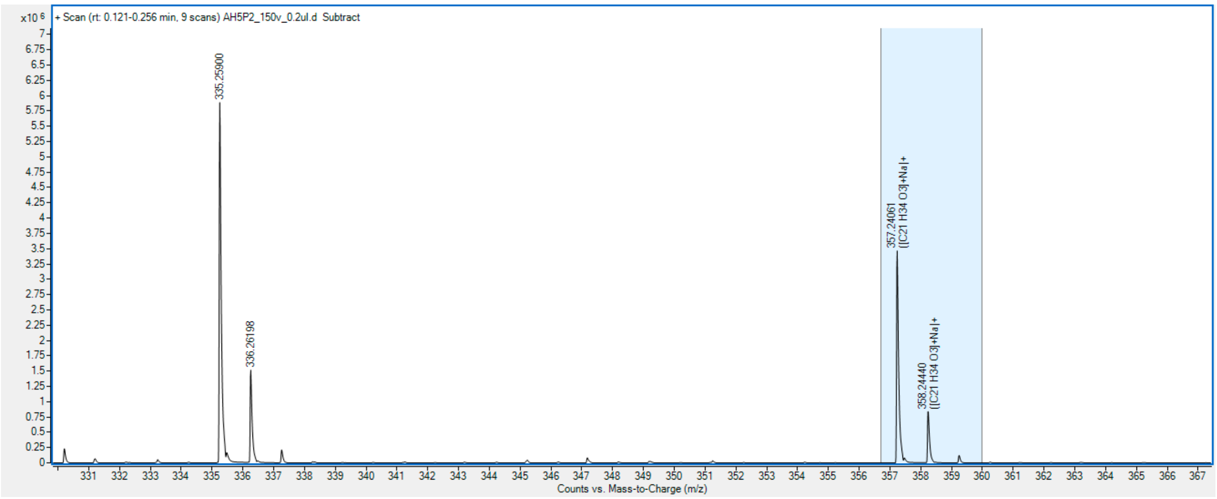

Table S4. Molecular formula generated from mass spectrometry of BH5 P2.

Formula	Species	m/z	Score	Diff(abs. ppm)	Mass
C21 H34 O3	(M+Na)+	357.24061	97.8	2.04	334.25079
